# The HDL receptor SR-BI is associated with human prostate cancer progression and plays a possible role in establishing androgen independence

**DOI:** 10.1186/s12958-015-0087-z

**Published:** 2015-08-07

**Authors:** David Schörghofer, Katharina Kinslechner, Andrea Preitschopf, Birgit Schütz, Clemens Röhrl, Markus Hengstschläger, Herbert Stangl, Mario Mikula

**Affiliations:** Institute of Medical Genetics, Medical University of Vienna, Währinger Strasse 10, 1090 Vienna, Austria; Institute of Medical Chemistry, Medical University of Vienna, Währinger Strasse 10, 1090 Vienna, Austria

**Keywords:** SCARB1, LDLR, Cholesterol, mTOR, Androgen synthesis

## Abstract

**Background:**

Human prostate cancer represents one of the most frequently diagnosed cancers in men worldwide. Currently, diagnostic methods are insufficient to identify patients at risk for aggressive prostate cancer, which is essential for early treatment. Recent data indicate that elevated cholesterol levels in the plasma are a prerequisite for the progression of prostate cancer. Here, we analyzed clinical prostate cancer samples for the expression of receptors involved in cellular cholesterol uptake.

**Methods:**

We screened mRNA microarray files of prostate cancer samples for alterations in the expression levels of cholesterol transporters. Furthermore, we performed immunohistochemistry analysis on human primary prostate cancer tissue sections derived from patients to investigate the correlation of SR-BI with clinicopathological parameters and the mTOR target pS6.

**Results:**

In contrast to LDLR, we identified SR-BI mRNA and protein expression to be induced in high Gleason grade primary prostate cancers. Histologic analysis of prostate biopsies revealed that 53.6 % of all cancer samples and none of the non-cancer samples showed high SR-BI staining intensity. The disease-free survival time was reduced (*P* = 0.02) in patients expressing high intra-tumor levels of SR-BI. SR-BI mRNA correlated with HSD17B1 and HSD3B1 and SR-BI protein staining showed correlation with active ribosomal protein S6 (RS = 0.828, *P* < 0.00001).

**Conclusions:**

We identified SR-BI to indicate human prostate cancer formation, suggesting that increased levels of SR-BI may be involved in the generation of a castration-resistant phenotype.

**Electronic supplementary material:**

The online version of this article (doi:10.1186/s12958-015-0087-z) contains supplementary material, which is available to authorized users.

## Background

Prostate cancer is one of the most common solid organ tumors in males. It is a slow growing type of tumor, but can potentially give rise to aggressive and metastasizing forms of cancer [1]. The risk for prostate cancer increases with consumption of a high fat, high cholesterol diet or the presence of hypercholesterolemia [2–4]. Very recently, it was shown that the accumulation of esterified cholesterol underlies the aggressiveness of human prostate cancer [5]. Cellular cholesterol is either synthesized by the cells themselves, or exogenous cholesterol is taken up and utilized by the cancer cells. Cholesterol uptake is mainly mediated by the high density lipoprotein receptor SR-BI and the low density lipoprotein receptor LDLR [6–9]. In normal tissue, SR-BI is expressed in the liver and in steroidogenic tissues, where cholesterol uptake is necessary for steroid hormone synthesis [10–13]. Notably, patients suffering from mutations in cla-1, the human homolog to SR-BI, display impaired steroid hormone synthesis [14]. There is evidence that SR-BI plays a role in prostate cancer development, specific antigen secretion and the viability of prostate cancer cells because it was shown that SR-BI-specific knockdown in LNCaP and C4-2 prostate carcinoma cells reduced PSA secretion and the viability of prostate cancer cell lines [15]. Therefore, this study aimed to evaluate the expression of receptors involved in cellular cholesterol uptake in clinical prostate cancer samples.

## Material and methods

### Bioinformatic analysis

For Gleason score analysis, the GSE2109 and GSE3933 datasets from the International Genomics Consortium Expression Project for Oncology were used [16]. The sample sizes were as follows: GSE2109, *n* = 56 (Gleason score ≤ 6 *n* = 20, Gleason score ≥ 7 *n* = 36), GSE3933, *n* = 58 for SR-BI (Gleason score ≤ 6 *n* = 24, Gleason score ≥ 7 *n* = 34) and *n* = 60 for LDLR (Gleason score ≤ 6 *n* = 24, Gleason score ≥ 7 n = 36). For metastasis analysis, the GSE35988, GSE3933 and GSE6919 datasets were used [16–19]. The sample sizes were as follows: GSE35988, *n* = 94 (primary site *n* = 59, metastasis *n* = 35), GSE3933, *n* = 68 for SR-BI (primary site *n* = 59, metastasis *n* = 9) and *n* = 68 for LDLR (primary site *n* = 61, metastasis *n* = 7) and GSE6919, *n* = 88 (primary site *n* = 64, metastasis *n* = 24). For Kaplan-Meier analysis, the GSE40272 dataset was used (sample size: *n* = 85) [20].

### Patient cohort and pathology

With institutional review board approval from the Medical University of Vienna (EK Nr: 1734/2014), tissue microarrays were obtained from US Biomax (Rockville, MD). All samples were formalin-fixed less than 10 min after surgery, paraffin embedded and assembled as cores with a diameter of 1.5 mm. Tissue sections were quality controlled and contained normal prostate tissue and prostate cancer tissue, representing different stages of disease progression. Each individual core was assigned to independent Gleason scoring and was reviewed by two board-certified pathologists.

### Immunohistochemistry

Prostate cancer tissue sections containing paraffin-embedded samples were melted for 20 min at 60 °C and rehydrated by subsequent incubation in Xylol, Isopropanol, 96 % Ethanol, 70 % Ethanol and 50 % Ethanol. Then, tissue sections were washed and heated to 120 °C in a pH 6.0 buffer or a pH 9.0 buffer (Dako, Glostrup, Denmark), depending on the antibody. After cooling to room temperature, samples were incubated with 1 % H_2_O_2_ (Sigma, St. Louis, MO) for 10 min. Afterwards, samples were permeabilized with 0.1 % Triton X-100 (Sigma) for 5 min. Then, sections were blocked with 2.5 % horse sera (Vector Laboratories, Burlingame, CA) for at least 20 min at room temperature. Subsequently, sections were incubated overnight at 4 °C with the primary antibodies directed against SR-BI (BD Transduction Laboratories™, Franklin Lakes, NJ), LDLR (Santa Cruz Biotechnology, Santa Cruz, CA) and pS6 (Cell Signaling Technology, Beverly, MA), diluted 1:200. For negative control staining, sections were incubated with matched isotope control antibodies instead of primary antibodies. Next, slides were washed and the corresponding secondary, biotinylated antibodies (Vector Laboratories) were added for 45 min at room temperature. After a washing step, sections were incubated for 30 min with Streptavidin-HRP (Leica, Wetzlar, Germany). For detection, tissue sections were incubated with AEC+ High Sensitivity Substrate Chromogen (Dako). Counterstaining with hematoxylin solution was performed according to Mayer (Carl Roth, Karlsruhe, Germany); tissue sections were mounted with Aquatex® (Merck Millipore, Billerica, MA).

### Evaluation of immunohistochemical staining

Evaluation of tissue sections was performed by two independent researchers who were blinded to the patients’ details. Immunostaining of the anti-SR-BI antibody was scored on at least duplicate tissues using the following arbitrary scale: no staining (0), low staining (1), medium staining (2) and high staining (3).

### Statistical analysis

Dot plots were generated with SPSS v21. The arithmetic mean of all samples is indicated by a line. Two-tailed *P*-values were calculated with unpaired (independent) t-tests in SPSS. *P*-values ≤ 0.05 were considered to be statistically significant. The significance of the difference between the variances of two samples was determined with Levene’s test. If the resulting *P*-value of Levene’s test was > 0.05, we assumed equal variances and adopted the output of the equal variance *t*-test as the *P*-value; if the resulting P-value of Levene’s test was ≤ 0.05, we assumed unequal variances and adopted the output of the unequal variance *t*-test as the *P*-value.

Scatter plots were generated with SPSS v21. Pearson correlation analysis was performed to calculate the P-values of the graphs.

For analysis of immunohistological staining results, the internet tool VassarStats (http://vassarstats.net/index.html) was used. Risk ratios were calculated using 2 × 2 contingency tables and the chi-square test was applied to determine the association of clinicopathological parameters with SR-BI expression.

Kaplan-Meier analysis and the log-rank test were performed using SPSS 21 to test the association of SR-BI and LDLR with disease-free survival time. A total of 85 samples were available for evaluation. For the analysis, samples were segregated into groups with SR-BI or LDLR levels above (42 samples) and below or equal (43 samples) to the median value.

## Results

### Evaluation of SR-BI and LDLR expression as markers for prostate cancer progression

**Fig. 1:**
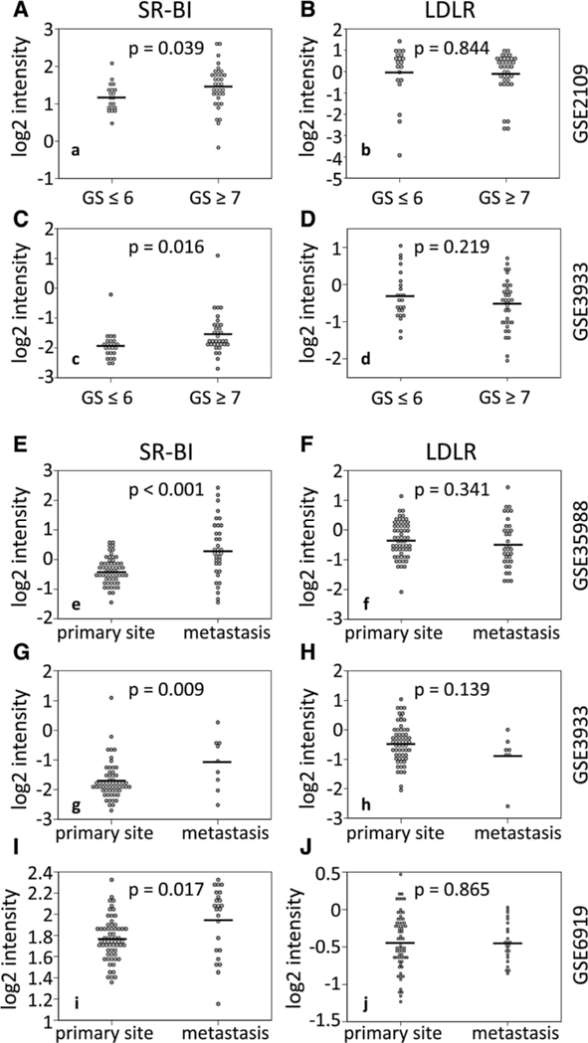
Expression of SR-BI and LDLR in clinical prostate samples. Differential expression of SR-BI and LDLR according to Gleason scoring (**a–d**). All samples were grouped according to Gleason score (GS) ≤ 6 and ≥ 7. Differential expression of SR-BI and LDLR in metastasizing and non-metastasizing prostate tissues (**e-j**). All samples were grouped into primary tumors (primary site) and metastasizing tumors (metastasis). *P*-values are presented within each graph. The arithmetic mean is given as a line within the dots. Data displayed as log 2 median-centered intensity

We analyzed SR-BI and LDLR mRNA expression in primary human prostate cancers from the Expression Project for Oncology and from a hallmark study by Lapointe *et al.* [16]. When comparing prostate cancer samples with high Gleason scores (equal to or higher than 7) and samples with low Gleason scores (equal to or lower than 6), SR-BI was more highly expressed in GSE2109 (*n* = 56, *P* = 0.039, Fig. 1a) and GSE3933 (*n* = 58, *P* = 0.016, Fig. 1c). LDLR was not increased in high Gleason score samples in GSE2109 (*n* = 56, *P* = 0.844, Fig. 1b) and in GSE3933 (*n* = 60, *P* = 0.219, Fig. 1d).

The most important parameter used to decide upon patient survival is the occurrence of metastasis. Therefore, we next determined the expression of SR-BI and LDLR in clinical prostate samples derived either from non-metastatic or metastatic prostate cancer. For this analysis, we investigated the dataset GSE35988, which contains benign and localized prostate cancer from radical prostatectomy as well as metastatic, castration-resistant prostate cancer obtained from rapid autopsy [17]. Furthermore, we included the datasets GSE6919 and GSE3933, which contain samples from the primary tumor site as well as metastasized prostate cancer samples from the liver, lung, kidney, adrenal gland or lymph nodes [16, 18, 19]. We identified an increased expression of SR-BI in metastatic prostate samples compared to non-metastatic prostate samples in GSE35988, GSE3933 and GSE6919 (*P* < 0.001, *P* = 0.009 and *P* = 0.017, respectively) (Fig. 1e, g, i,). Contrary to SR-BI, LDLR expression was not increased in metastatic prostate samples compared to non-metastatic prostate samples in GSE35988, GSE3933 and GSE6919 (*P* = 0.341, *P* = 0.139 and *P* = 0.856, respectively) (Fig. 1f, h, j).

### Association of clinicopathological parameters with SR-BI expression

**Fig. 2:**
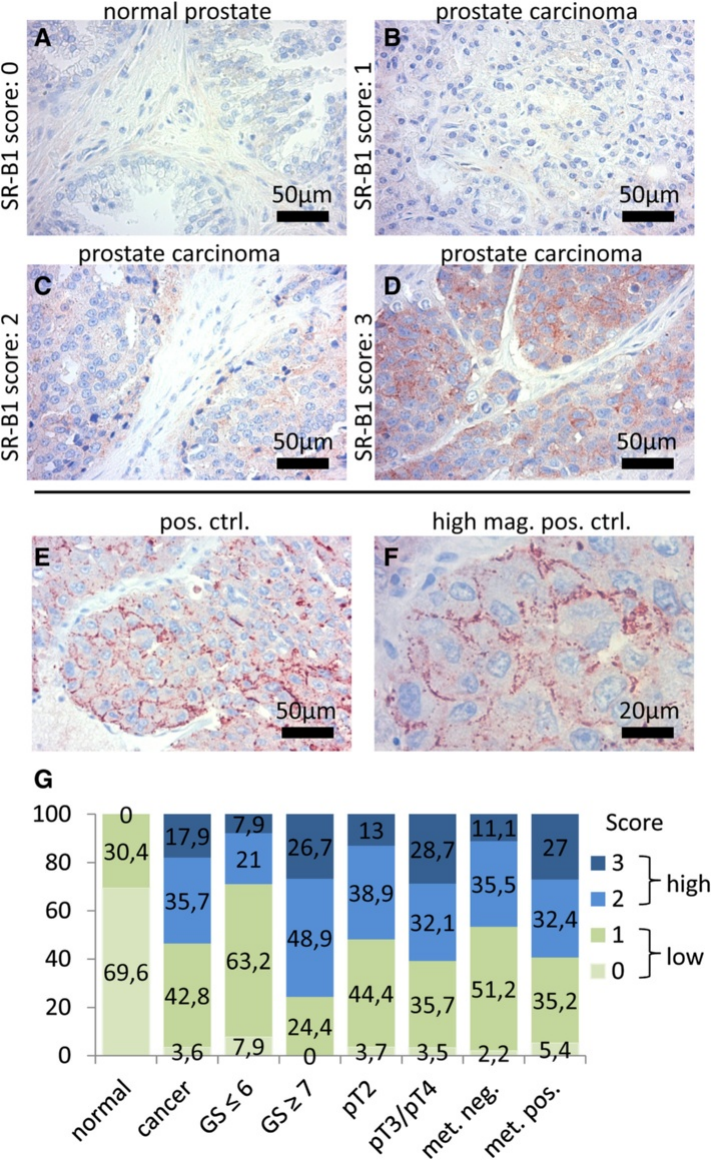
Immunohistochemical staining of prostate tissue and control tissue for SR-BI expression. Each panel shows representative prostate samples scored for staining intensity as follows: 0 for negative (**a**), 1 for low (**b**), 2 for moderate (**c**) and 3 for high (**d**). Liver sections were used as positive controls (pos. ctrl.) in standard magnification (**e**) and in high magnification (high mag.), demonstrating SR-BI localization in the outer cell membrane of hepatocytes (**f**). The overall scoring distribution for SR-BI staining intensity in different clinicopathological groups (**g**). Scores of 0 and 1 represented “low” expression and are shown in green colors, whereas scores of 2 and 3 represented “high” expression and are shown in blue colors. Clinicopathological groups are plotted on the x-axis: non-cancer and cancer, Gleason score (GS) ≤ 6 and ≥ 7, pathologic tumor stage 2 (pT2) and pathologic tumor stage 3/4 (pT3/4), metastasis negative (met. neg.) and metastasis positive (met. pos.)

To confirm findings from mRNA expression studies, we assessed SR-BI protein expression in normal prostate tissue and prostate cancer samples derived from patients with known TNM status. A total of 106 biopsy cores were independently assigned to Gleason scoring. Cores were subjected to immunohistochemical staining for SR-BI and afterwards analyzed for their staining intensity. Table 1 shows the clinicopathological characteristics of the cohort studied. Samples were classified into non-cancer and cancer samples. Cancer samples were further characterized by Gleason score, pathologically classified tumor stage (pT) and metastasis. Nearly half of all cancer samples showed a Gleason score equal to or above 7. Furthermore, about a third of all cancer samples showed advanced tumor stages (pT3/4), and nearly half of them were positive for metastasis. Representative cores for different staining intensities of SR-BI and their respective scores are shown in Fig. 2 (a–d). We tested the specificity of our antibodies by staining human liver sections (Fig. 2e and f). The distribution of the results for the whole tissue collective is shown in Fig. 2g. On the basis of a binary classification system for low (score 0 and score 1) and high (score 2 and score 3) SR-BI staining intensities, we evaluated associations between SR-BI and the presence or absence of clinicopathological parameters. Notably, out of 23 normal prostate samples, none showed high staining results (score 2 and score 3), and among all cancer samples, approximately 53.6 % showed high staining for SR-BI.

**Table 1:** Frequencies of clinicopathological characteristics

Characteristics		Sample size
Type	Non-cancer	23
	Cancer	83
Gleason score	≤6	38
	≥7	45
Tumor stage	pT2	54
	pT3/pT4	29
Metastasis	Negative	45
	Positive	38

As shown in Table 2, we identified high SR-BI expression to be associated with the presence of prostate cancer when compared to non-cancer prostate tissue (risk ratio = 2.154, *P* < 0.0001). Furthermore, we identified an association of high SR-BI score with a Gleason score equal to or higher than 7 (risk ratio = 2.907, *P* < 0.0001).

**Table 2:** Evaluation of the prognostic significance of SR-BI staining intensity

Variable	*P*-value	Risk ratio	95 % Confidence interval
Cancer vs. non-cancer	<0.0001	2.128	1.693–2.675
GS ≥ 7 vs. GS ≤ 6	<0.0001	2.907	1.673–5.050
pT3/pT4 vs. pT2	0.371	1.269	0.739–2.182
Met. pos. vs. met. neg.	0.207	1.351	0.837–2.182

**Fig. 3:**
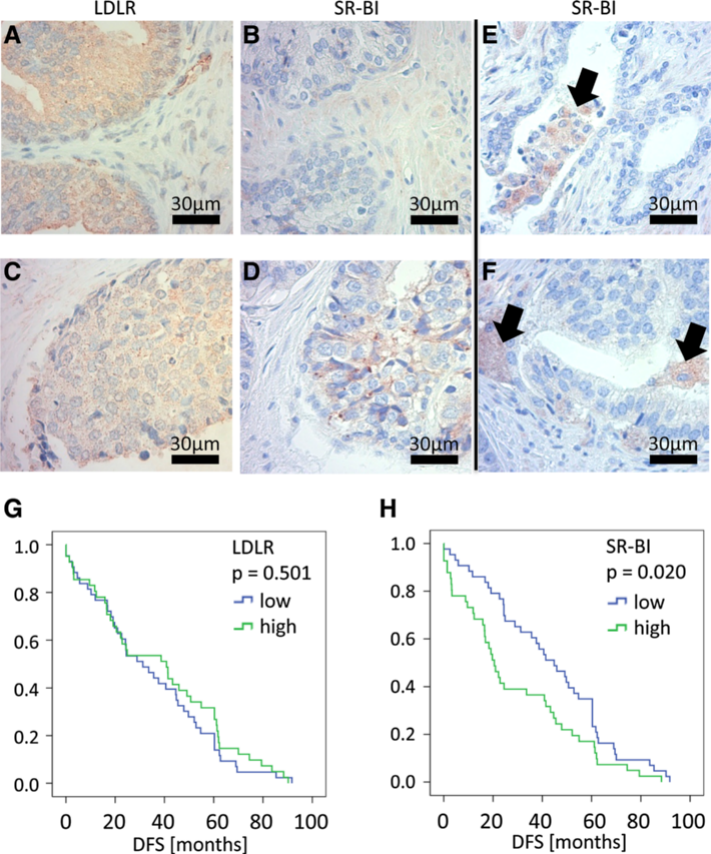
Lipoprotein receptor expression patterns in prostate cancer (high grade and low grade). Histologic staining for LDLR and SR-BI in selected patients. Tissue derived from a 27-year-old patient diagnosed with prostate hyperplasia was stained for LDLR (**a**); a consecutive area of the same tumor was stained for SR-BI (**b**). Tissue derived from a 75-year-old patient diagnosed with prostate cancer T2N1M1c and Gleason score 5 + 4 was stained for LDLR (**c**); a consecutive area of the same tumor was stained for SR-BI (**d**). Tumor biopsies from a 72-year-old patient diagnosed with prostate cancer T2N0M0 and Gleason score 3 + 3 were analyzed for SR-BI expression (**e** and **f**). Black arrows indicate clusters of cells strongly positive for SR-BI. Kaplan Meier analysis of LDLR and SR-BI expression in GSE40272 (**g–h**). High LDLR expression had no effect on disease-free survival time (**g**). High SR-BI expression was associated with decreased disease-free survival time (**h**). Green = high expression (high), blue = low expression (low), DFS = disease-free survival time. *P*-values of the log-rank test are presented within each graph

Because SR-BI expression showed an association with prostate cancer differentiation, we also tested for LDLR expression on selected sections with either low or high SR-BI staining intensity (Fig. 3a–d). Our case study showed that LDLR was constitutively expressed in prostate tissue, with lower expression levels in high-grade cancer samples. Interestingly, we also observed cases of low-grade prostate cancer, which displayed high SR-BI expression in a subpopulation of cells showing signs of tissue invasion (Fig. 3e and f). Cancer cells of this subpopulation either grew detached from the primary tumor, floating in the remaining glands (Fig. 3e), or separated from the solid tumor mass, infiltrating the surrounding tissue (Fig. 3f).

To assess whether SR-BI and LDLR had any influence on the clinical outcome of patients, we chose to evaluate the disease-free survival time in relation to SR-BI and LDLR expression. Therefore, we performed Kaplan-Meier analyses of the dataset GSE40272, which contained mRNA expression data on prostate tissue samples from men who underwent radical prostatectomy [20]. The disease-free survival time was defined as the time between surgery and the recurrence of disease (serum PSA > 0.1 ng/ml on two consecutive measurements after surgery). We identified samples with low SR-BI expression to have a significantly better survival outcome compared to samples with high SR-BI expression (p = 0.02, Fig. 3h). By contrast, there was no significant difference in disease-free survival time between samples with low LDLR expression and samples with high LDLR expression (Fig. 3g).

### Correlation of SR-BI with androgen-synthesizing enzymes and the mTOR pathway

**Fig. 4:**
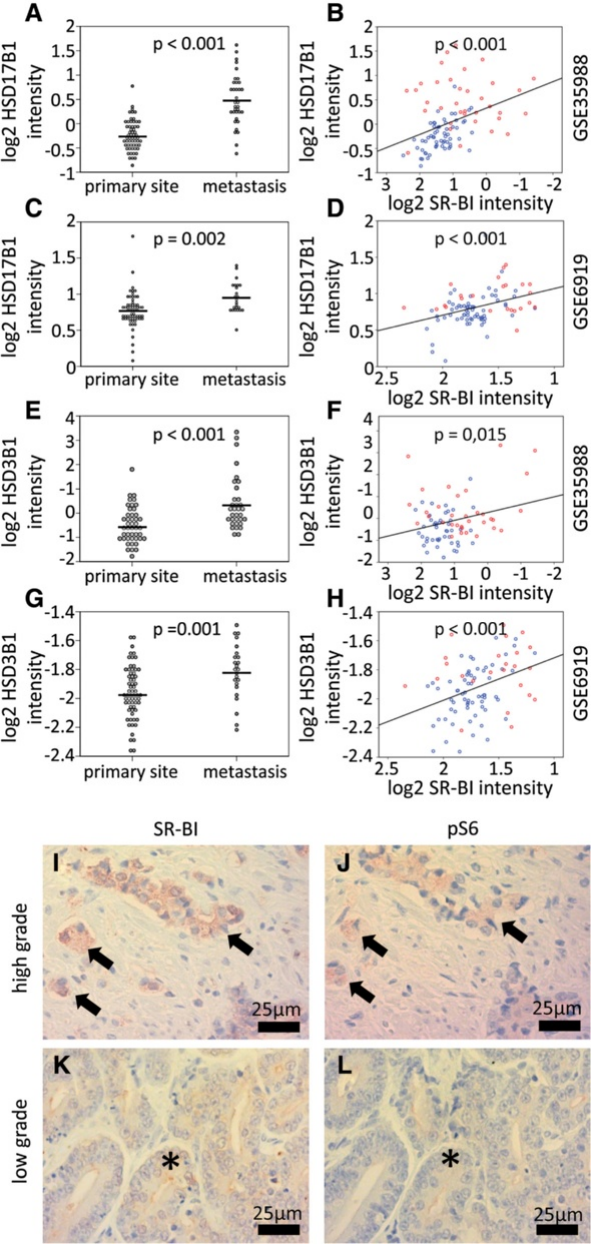
Correlation of SR-BI with androgen-synthesizing enzymes and the mTOR pathway. Differential expression of HSD17B1 and HSD3B1 in primary tumors (primary site) and metastasizing tumors (metastasis) (**a, c, e, g**). The arithmetic mean is given as a line within the dots and the *P*-values of *t*-test analysis is given within each graph. Scatter plots correlating SR-BI expression with HSD17B1 and HSD3B1 in prostate cancer (**b, d, f, h**). P-values of Pearson correlation analyses and regression lines are presented within each graph. Red dots represent metastatic and blue dots represent non-metastatic prostate cancer. Analysis of the co-occurrence of SR-BI and ribosomal protein S6 phosphorylation (**i–l**). A representative high grade prostate cancer is shown with staining for SR-BI (**i**) and for S6 phosphorylation at serine 240 and 244 in a consecutive area of the same tumor (**j**). A representative low grade prostate cancer is shown with staining for SR-BI (**k**) and for S6 phosphorylation at serine 240 and 244 in a consecutive area of the same tumor (**l**). pS6 = ribosomal protein S6 phosphorylation at serine 240 and 244

Because SR-BI mediates the selective uptake of cholesterol, which can be used for steroidogenesis, we analyzed enzymes that participate in androgen synthesis. We identified the β-hydroxysteroid-dehydrogenases HSD17B1 and HSD17B3 to be significantly up-regulated in metastatic compared to non-metastatic prostate cancer; they also correlated with the intensity of SR-BI expression (Fig. 4a–h).

Mechanistic studies have shown that mTOR signaling can mediate androgen independence [21]. Therefore, we further assessed the association of SR-BI expression with serine phosphorylation of ribosomal protein S6 at position 240 and 244. A total of 22 biopsy cores were subjected to immunohistochemical staining for pS6, and adjacent sections from the same patients were simultaneously subjected to SR-BI staining. Representative histologic staining for high grade and low grade prostate carcinoma samples is shown in Fig. 4 (i–l). After the pS6 and SR-BI staining, the samples were analyzed for their staining intensity. Representative cores for different staining intensities of pS6 and their respective scores are shown in Additional file 1: Figure S1 (A–D). Spearman correlation analysis revealed a significant positive correlation of SR-BI and pS6 (R = 0.828, *p* < 0.001).

## Discussion

Prostate cancer is the most commonly diagnosed malignancy in men and it has the potential to progress to a metastatic and highly aggressive form of cancer, which is still difficult to cure. Therefore, it is of profound importance to identify markers that allow the prediction of prostate cancer progression to its aggressive metastatic form. Recent studies suggest that cholesterol plays a major role in prostate cancer [15, 22–24]. In human cells, cholesterol uptake is mainly based on two pathways: receptor-mediated endocytosis by the LDL receptor and selective lipid uptake by SR-BI [6–8]. Here, we show an association of prostate cancer malignancy with the expression of the HDL receptor SR-BI. Our analysis of 306 clinical prostate samples for mRNA and 106 prostate tissue biopsy cores for protein expression identified significantly higher SR-BI expression in high Gleason grade versus low Gleason grade prostate cancer samples. Furthermore, our analysis of gene expression profiles identified significantly higher SR-BI mRNA expression in metastatic compared to non-metastatic prostate cancer. Strikingly, we further discovered an association of SR-BI expression with disease-free survival time in a cohort of 85 clinical prostate samples. Previous studies already suggested a connection of SR-BI expression with prostate cancer: the knockdown of SR-BI has been shown to reduce PSA levels and the viability of prostate cancer cells *in vitro* [15]. Moreover, SR-BI was found to be significantly up-regulated with progression to lethal castration-resistant prostate cancer (CRPC) in an LNCaP xenograft mouse model [25], while androgen-tolerant LNCaP cells *in vitro* did not show SR-BI up-regulation [26]. SR-BI has further been linked to nasopharyngeal cancer [27], colorectal cancer [28], ovarian cancer [28] and most notably breast cancer [29, 30], a tumor strongly dependent on the synthesis of sexual hormones. Furthermore, it was demonstrated that mutations of SR-BI affected the proliferation and apoptosis of the breast cancer cell line MCF-7 [30]. Knockdown of SR-BI was shown to inhibit proliferation and migration in breast cancer, and SR-BI knockdown also caused a decrease of tumor growth in MDA-MB231 and MCF-7 breast cancer cells *in vivo* when injected into nude mice [29].

The mTOR pathway plays a key role in the regulation of cellular growth and metabolism [31, 32]. Together with raptor and LST8, mTOR forms a complex called mTORC1 (mTOR complex 1), which acts by activating the ribosomal protein S6 through the protein kinase S6K1 [31, 32]. It is further known that mTORC1 influences cholesterol synthesis and uptake via the SREBP pathway [33–35]. Recently, it was shown that the inhibition of mTOR via rapamycin down-regulates SR-BI expression in human umbilical vein endothelial cells, indicating a direct connection between mTOR activation and SR-BI expression [36]. Further, it is known that mTOR plays a crucial role in the progression of prostate cancer to CRPC by influencing the androgen signaling pathway [37, 38]. According to our results, pS6 expression significantly correlates with SR-BI expression, which suggests the regulation of SR-BI by mTORC1 in prostate cancer.

To our knowledge, SR-BI has not been thoroughly studied in clinical samples of prostate cancer, and our findings on the mRNA and protein expression of SR-BI can contribute substantially to our understanding of prostate cancer progression. This study demonstrates the high expression of SR-BI in de-differentiated and metastasized prostate cancer, which almost always acquires resistance to androgen depletion. Therefore, we suggest that increased levels of SR-BI are involved in the transport of cholesterol into the tumor cell. This uptake of cholesterol could be exploited by the cancer cell to up-regulate its androgen synthesis. We observed the up-regulation of 3β- and 17β-hydroxysteroid dehydrogenases, which may play an important yet unclear role in intra-tumoral androgen synthesis [39, 40]. This process may contribute to the generation of castration-resistant prostate cancer. Therefore, pharmacologic inhibition of the HDL receptor might represent a way to inhibit prostate cancer progression. We suggest that SR-BI may be a valuable target for prostate cancer therapy; therefore, we strongly recommend that further studies investigate the role of SR-BI during prostate cancer progression.

## Conclusions

Here we have shown that the HDL receptor SR-BI can be induced during the course of prostate cancer formation and progression. Intra-tumor expression was associated with an increase in Gleason scoring and also metastatic prostate tissue showed SR-BI up-regulation compared to primary tumor tissue. Importantly, we identified positive correlation of SR-BI expression with expression of androgen synthesizing enzymes and mTOR activation.

## Additional file

Additional file 1: Figure S1. Immunohistochemical staining of prostate tissue for ribosomal protein phosphorylation at serine 240 and 244. Each panel shows representative prostate samples scored for staining intensity as follows; 0 for negative (A), 1 for low (B), 2 for moderate (C) and 3 for high levels of pS6 (D). pS6 = ribosomal protein S6 phosphorylation at serine 240 and 244. (JPEG 537 kb)
