# Supplementary figures and images for: The HDL receptor SR-BI is associated with human prostate cancer progression and plays a possible role in establishing androgen independence
Source: Reprod Biol Endocrinol. 2015 Aug 7;13:88. doi: 10.1186/s12958-015-0087-z (PMC4528807; doi:10.1186/s12958-015-0087-z)

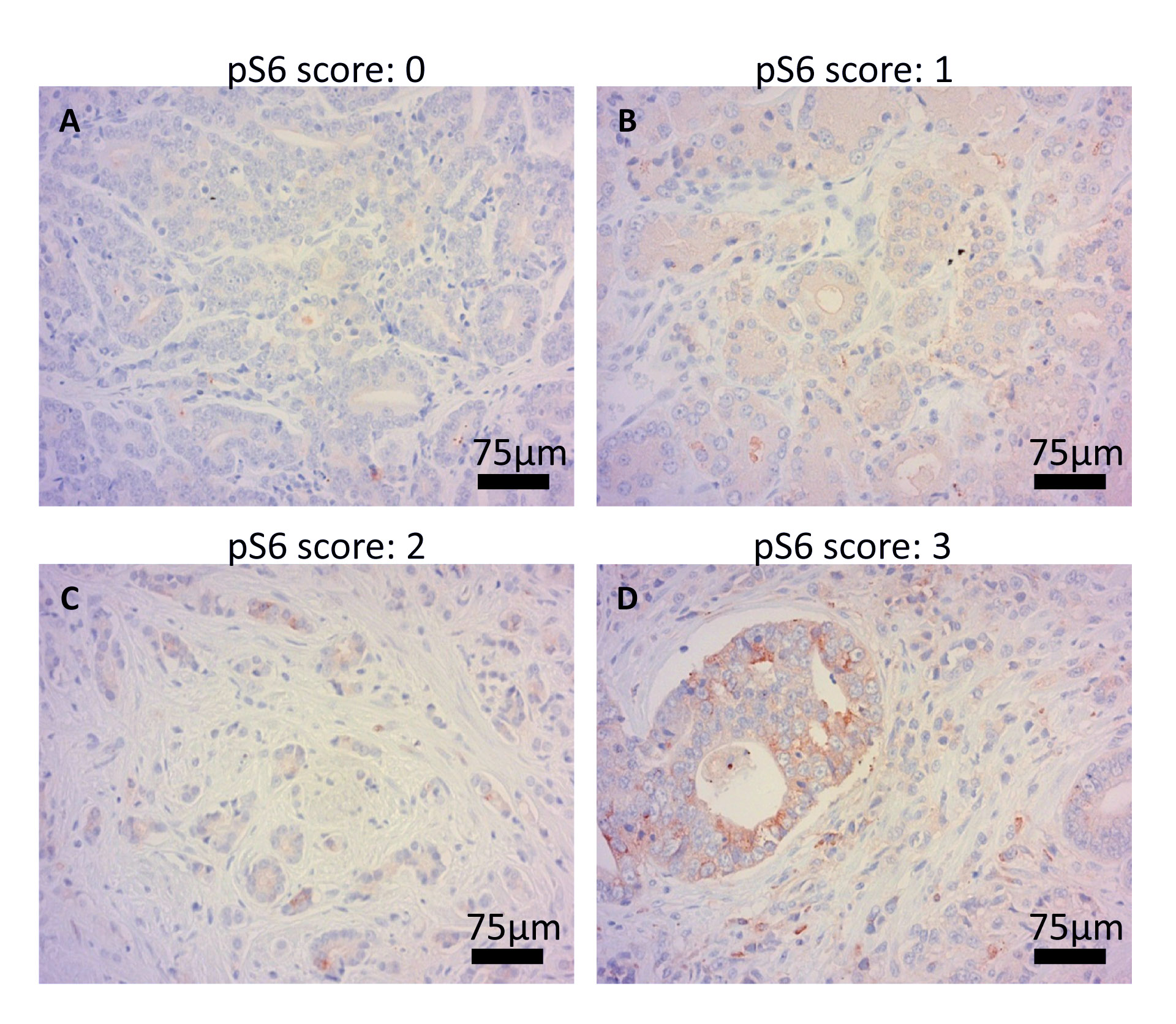

Supplement: Additional file 1: Figure S1. — Immunohistochemical staining of prostate tissue for ribosomal protein phosphorylation at serine 240 and 244. Each panel shows representative prostate samples scored for staining intensity as follows; 0 for negative (A), 1 for low (B), 2 for moderate (C) and 3 for high levels of pS6 (D). pS6 = ribosomal protein S6 phosphorylation at serine 240 and 244. (JPEG 537 kb) [file 12958_2015_87_MOESM1_ESM.jpg]
